# Diseases of the Eye

**Published:** 1898-04

**Authors:** 


Diseases of the Eye. By Edward Nettleship, F. R. C. S., Ophthalmic Surgeon at St. Thomas' Hospital, London; Surgeon to the Royal London (Moorfields) Ophthalmic Hospital. Revised and edited by W. T. Holmes Spicer, M. A., M. B., F. R. C. S., Ophthalmic Surgeon to the Metropolitan Hospital and to the Victoria Hospital for Children. Fifth American from the sixth English edition. With a Supplement on Color-Blindness, by William Thompson, M. D., Emeritus Professor of Ophthalmology in the Jefferson Medical College of Philadelphia. With two colored plates and 161 engravings. Duodecimo, pp. 528. Philadelphia and New York: Lea Brothers & Co. 1897.

Nettleship's compendium on diseases of the eye is now in its fifth American and sixth English edition. This fact alone is proof positive of its merits, as judged by the English-speaking profession on both sides of the Atlantic. It supplies a need that does not seem to be met by any other handbook, and has steadily risen in esteem from its first appearance, nearly twenty years ago, up to the present time. It serves the double purpose, so far as it is possible in a single volume, of meeting the requirements of the student by not neglecting the elements of ophthalmology, and of satisfying the needs of the practitioner by clearly enlightening him on subjects pertaining to daily practice.

Each successive edition has been carefully revised and all the essentials of progress in ophthalmology up to the time of issue have been incorporated. Heretofore this has been done by Mr. Nettleship himself, but the revision for the present edition has been entrusted to Mr. Holmes Spicer, a most able ophthalmologist of London and by the American publishers to an equally eminent American authority. The result is that the book not only represents English thought and practice, but also that of American ophthalmologists who differ somewhat from their English confrères on certain subjects. The additions of the American reviser are not numerous and some of them might have been more valuable had he enlarged them somewhat, especially the one on heterophoria.

New life has again been infused in this valuable handbook and it deserves even greater favor than it has ever received before.

